# Treatment of hospital-acquired pneumonia with multi-drug resistant organism by *Buzhong Yiqi* decoction based on *Fuzheng Quxie* classical prescription: study protocol for a randomized controlled trial

**DOI:** 10.1186/s13063-019-3927-x

**Published:** 2019-12-30

**Authors:** Dong Deng, Zhenyi Chen, Liyang Jia, Jianhong Bu, Miaoqing Ye, Lihua Sun, Yun Gen, Wen Zhang, Gang Chen, Bangjiang Fang

**Affiliations:** 1 LongHua Hospital Shanghai University of Traditional Chinese Medicine, NO.725 Wanping South Road, Xuhui District, Shanghai, 200032 China; 2 The Second Clinical Medical College, Henan University of Traditional Chinese Medicine, NO.6 Dongfeng Road, Zhengzhou, 450046 Henan China; 3 Shanghai Municipal Hospital of Traditional Chinese Medicine, No. 274 Zhijiang Middle Road, Jing’an District, Shanghai, 200071 China; 4 Department of Liver Disease, Shaanxi Provincial Hospital of Traditional Chinese Medicine, NO.4 xihuamen, Lianhu District, Xi’an, 710003 Shaanxi China; 5 Shanghai Seventh People’s Hospital, NO.358 Datong Road, Gaoqiao, Pudong New District, Shanghai, 200137 China

**Keywords:** Hospital acquired pneumonia caused by multi-drug resistant organisms, *Buzhong Yiqi* decoction, Efficacy, Multicenter, Randomized controlled clinical study

## Abstract

**Background:**

Drug resistance in China is becoming a more and more serious issue. Infection by drug-resistant bacteria has become a major disease that seriously threatens the health of Chinese people and affects national medical finance. Therefore, it is of great scientific and clinical significance to actively carry out research on the prevention and treatment of infections by multi-drug resistant organisms (MDRO). Previous studies by the authors suggested that patients with hospital-acquired pneumonia caused by MDRO mostly showed the pathological state of “insufficient healthy *Qi* and internal accumulation of pathogenic *Qi*” and “acute deficiency syndrome” mainly characterized by *Qi* deficiency. *Buzhong Yiqi* decoction is a famous classic prescription in traditional Chinese medicine (TCM) for treating internal damage fever. This study intends to provide an evidence-based rationale for *Buzhong Yiqi* decoction in treating MDRO hospital-acquired pneumonia by conducting a multi-center randomized controlled clinical study.

**Methods/design:**

This study is designed to be a multi-center randomized controlled study in which patients are assigned randomly into control (standard therapy) and trial (standard therapy plus *Buzhong Yiqi* decoction) groups. The patients will be selected from the emergency department and the ICU inpatient department of five study sites and will all be diagnosed with MDRO hospital-acquired pneumonia and meet the inclusion criteria. Forty patients are to be enrolled in each study site, resulting in a total of 200 patients in the study. The treatment course is 28 days.

**Discussion:**

In this study: (1) the theory of “acute *Qi* deficiency” in MDRO hospital-acquired pneumonia is put forward for the first time, and the basic theories of TCM are further improved; (2) a multi-center randomized controlled clinical study will be performed for the first time with *Buzhong Yiqi* decoction, the classic prescription for reinforcing healthy *Qi* and eliminating pathogenic *Qi*, providing a reliable evidence-based rationale for the treatment of MDRO pulmonary infection with TCM; (3) the clinical application and modern disease spectrum of *Buzhong Yiqi* decoction is expanded, and the scientific notion of “treating different diseases with the same method” is enriched further.

**Trial registration:**

China Clinical Trial Registry, ChiCTR1900022429. Registered on April 11, 2019. http://www.chictr.org.cn/listbycreater.aspx.

## Background

Clinical studies on hospital-acquired pneumonia (HAP) and/or ventilator-associated pneumonia (VAP) have detected multi-drug-resistant organisms (MDROs) in most respiratory secretions from patients in intensive care units (ICUs), with the isolation rate increasing year on year [1–4]. In recent years, the national bacterial resistance surveillance report has revealed that MDROs have, to varying degrees, serious epidemic characteristics in different provinces of China. The increasing social burden of MDRO infections arises from adverse clinical outcomes and aggravated economic and medical burdens [5–7].

HAP caused by MDRO infection is a common and major clinical disease, especially in emergency departments and ICUs [8]. According to its clinical manifestations, the disease can generally be classified as “pulmonary fever” in traditional Chinese medicine. Our previous clinical studies found that HAP caused by MDROs, due to the underlying and excessive pathogenic factors prevailing over the normal resistance of the body, first showed the signs of *Qi* deficiency, especially weakness of the spleen and stomach, giving rise to a pathological state of mainly “acute *Qi* deficiency”. Combining with theories from traditional Chinese medicine (TCM), we innovatively put forward a theory of “acute deficiency syndrome” in critical and severe cases of HAP caused by MDRO infection. Based on studies by other scholars on TCM syndrome types of MDRO HAP [9–12], we believe that the core pathogenesis of MDRO HAP is “insufficient healthy *Qi* and internal accumulation of pathogenic *Qi*”, in which “acute *Qi* deficiency syndrome” is critical.

*Buzhong Yiqi* decoction was proposed in *On Spleen and Stomach* by Li Dongyuan, one of the four greatest medical scientists in the Jin and Yuan Dynasties. It is a famous classical prescription in TCM and also a typical representative prescription in treating internal damage fever. According to TCM, “Pathogenic *Qi* cannot invade the body with sufficient healthy *Qi* inside” and disease occurs when healthy *Qi* is weak. Supplementing pectoral *Qi* is the method to cure the root cause, and abundance of pectoral *Qi* depends on the lung and spleen. Li’s *Buzhong Yiqi* decoction was formulated considering the methods of “treating impairment with supplementing” and “treating overstrain with warming” in *The Yellow Emperor’s Canon of Internal Medicine*, pioneering the school of “relieving great fever with drugs sweet in taste and warm in property”. This prescription consists of *Radix Astragali seu Hedysari*, *Radix Ginseng*, *Radix Glycyrrhiza*, *Rhizoma Atractylodis Macrocephalae*, *Radix Angelicae Sinensis*, *Pericarpium Citri Reticulatae*, *Rhizoma Cimicifugae*, and *Radix Bupleuri*. In the prescription, *Radix Astragali seu Hedysari* is the sovereign medicinal that invigorates *Qi*, *Radix Ginseng* is the minister medicinal that powerfully tonifies the primordial *Qi*, nourishes the lung, and invigorates the spleen, *Rhizoma Atractylodis Macrocephalae* invigorates the spleen, *Radix Angelicae Sinensis* regulates the blood, *Pericarpium Citri Reticulatae* regulates *Qi*, and prepared *Radix Glycyrrhiza* helps *Radix Ginseng* and *Radix Astragali seu Hedysari* rectify *Qi*; *Rhizoma Cimicifugae* and *Radix Bupleuri* have the function of eliminating pathogens and raising the clear yang. The whole formula supplements and clears in that it not only tonifies the middle and replenishes *Qi*, but also unblocks [13–16].

Clinical pharmacological studies with *Buzhong Yiqi* decoction have demonstrated that it enhances phagocytosis of the reticuloendothelial system, promotes the non-specific immune function of the body, and has a remarkable inhibitory effect on multi-drug-resistant *Staphylococcus aureus* [17, 18]. This recipe can effectively alleviate inflammatory reactions and boost immunity of the body. Results from previous studies by the authors on *Buzhong Yiqi* decoction for the treatment of HAP, including that caused by MDROs, showed that TCM could significantly relieve the inflammatory reactions of patients, improve clearance of the organisms, shorten the time of mechanical ventilation, and increase the success rate of weaning from the ventilator and had good clinical efficacy. Similar clinical efficacy was observed in other related studies on *Buzhong Yiqi* decoction for the treatment of HAP [19–22]. Therefore, on the basis of previous studies, this study intends to provide an evidence-based rationale and guidance for the clinical treatment of MDRO HAP, further elucidate the scientific notion of “treating different diseases with the same method”, and expand the modern disease spectrum of *Buzhong Yiqi* decoction by performing a multi-center randomized controlled clinical study with *Buzhong Yiqi* decoction (a classical formula for reinforcing healthy *Qi* and eliminating pathogenic *Qi*) in the treatment of MDRO HAP.

## Methods/design

### Objective

We propose a multi-center randomized controlled clinical study to demonstrate the efficacy and safety of *Buzhong Yiqi* decoction in the treatment of MDRO HAP and to explore the possible mechanism, provide a reliable evidence-based rationale for the treatment of MDRO pulmonary infection with TCM, further elucidate the scientific notion of “treating different diseases with the same method”, and expand the modern disease spectrum of *Buzhong Yiqi* decoction. We do this to open up new ideas and methods for MDRO prevention and treatment strategies and formulate TCM diagnosis and treatment regimens for pulmonary infections caused by MDROs in clinical practice.

### Design

**Fig. 1:**
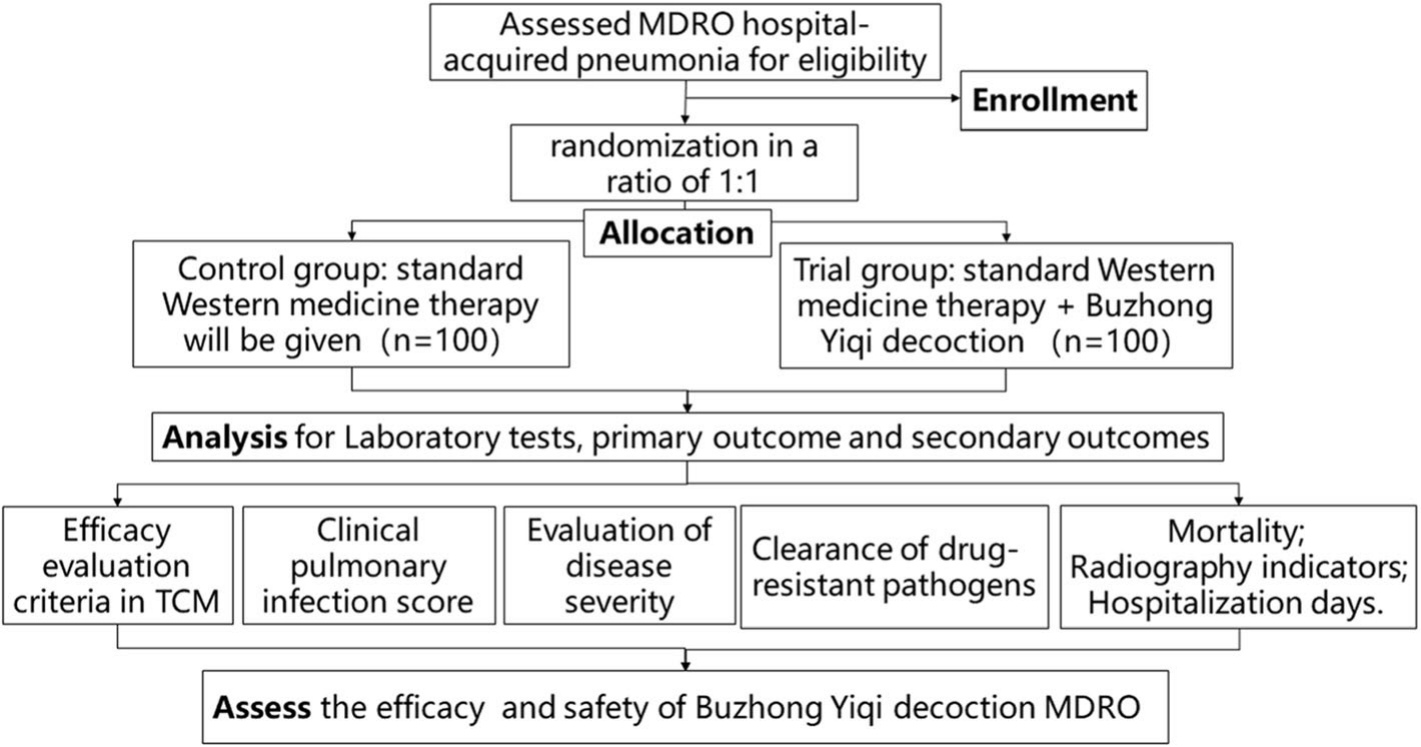
Flow chart of the study

This study is a multi-center randomized controlled study in which patients will be assigned randomly into control and trial groups. The patients will be selected from the emergency department and the ICU inpatient department of five study sites after being diagnosed with MDRO HAP and meeting the inclusion criteria. The flow chart of the study is shown in Fig. 1. The date of study initiation was July 1, 2018 and the date of study completion is June 30, 2021. The Standard Protocol Items: Recommendations for Interventional Trials (SPIRIT) Checklist can be found in Additional file 1.

### Participants

From July 1, 2018 to May 31, 2021, patients will be selected from those treated in the emergency department and the ICU inpatient department of the five study sites, namely Longhua Hospital Shanghai University of Traditional Chinese Medicine, Shanghai Integrated Traditional Chinese and Western Medicine Hospital, Shanghai Seventh People’s Hospital, Shanghai Baoshan Integrated Traditional Chinese and Western Medicine Hospital, and Shanghai Pudong New Area Public Interest Hospital. All patients are diagnosed with MDRO HAP and meet the inclusion criteria. Forty patients are to be enrolled in each study site, resulting in a total of 200 patients in the study.

### Inclusion criteria

The eligibility criteria are as follows: (1) patients meet the diagnostic criteria of Western medicine; (2) etiological diagnosis meets the MDRO criteria; (3) aged 40–85 years; (4) patients have signed the informed consent form; (5) Patients also meeting the following criteria as Exclusion criteria, withdrawal/trial discontinuation/drop-out criteria are included into observation.

### Exclusion criteria

The exclusion criteria are as follows: (1) severe primary or malignant disease of the heart, brain, liver, kidney, or other organs or systemic disease at an acute or progressive stage; (2) surgery within 2 months accompanied by surgical site infection; (3) patients who have been hospitalized less than 72 h or who die within 72 h of hospitalization; (4) psychiatric patients and pregnant women; (5) active tuberculosis; (6) a history of allergy to on-treatment drugs; (7) recent drug and alcohol abuse; (8) patients who receive no other antibiotics or fail to respond to other antibiotics within 3 months prior to the clinical trial.

### Withdrawal/trial discontinuation/drop-out criteria

The rejection and withdrawal criteria are: (1) not meeting the inclusion criteria of the study; (2) incomplete clinical data obtained after inclusion and it is impossible to perform further clinical statistical analyses; (3) subjects experience severe adverse events/reactions related to the treatment regimen and the investigator considers it necessary to withdraw them from the trial; (4) during the trial, the patient’s condition continues to deteriorate and dangerous events are likely to occur such that the investigator considers it necessary to withdraw them from the clinical trial; (5) non-specified combination of drugs, especially those that have a great impact on the observational drug and affect efficacy and safety assessment; (6) patients who withdraw during the trial at their own discretion—all patients who complete the informed consent form and are eligible to enter the trial at screening, no matter when and why they withdraw, are classified as drop-out cases as long as they do not complete the observational period specified in the protocol; (7) poor treatment compliance that affects determination of efficacy and safety.

### Ethics

This trial is conducted in accordance with the *Helsinki Declaration* and Chinese Good Clinical Practice and relevant regulations and has been approved by the Medical Ethics Committee of Longhua Hospital Shanghai University of Traditional Chinese Medicine. To protect the privacy of subjects, data processing is performed anonymously and written informed consent is obtained from each patient before initiation of the clinical trial.

### Randomization and allocation

In each study site, patients with MDRO HAP that fulfill the inclusion criteria and do not meet any of the exclusion criteria are assigned to the trial group or control group according to the corresponding random numbers extracted in the order at visit. Each study site will enroll 40 patients for a total of 200 over the five study sites. A random number table generated using Statistical Analysis Software (SAS), version 9.2, using the randomization method, will be used to assign the participants in a ratio of 1:1, with 100 patients each in the trial group and control group. A researcher will generate the allocation sequence and enroll participants and the clinical researchers will assign participants to interventions. The clinical researchers at each center will provide packaged drugs to the participants according to the randomization number on the packaging; the code labeling will conform to the principles of GCP. The statistician will uncover the blinding as necessary.

### Interventions

The treatment course is 28 days.

The control group will be given standard Western medicine therapy, including: routine monitoring of vital signs; blood, arterial blood gas, and biochemical analysis; coagulation monitoring; step-down antimicrobial therapy; handle the respiratory secretions; mechanical ventilation; liquid nutrient support; maintenance of blood sugar, acid base, and electrolyte balance; correct coagulation function; etc.

The trial group will receive standard Western medicine therapy plus *Buzhong Yiqi* decoction (*Radix Astragali seu Hedysari* 45 g, *Radix Codonopsis* 15 g, fried *Rhizoma Atractylodis Macrocephalae* 15 g, prepared *Radix Glycyrrhiza* 15 g, *Radix Angelicae Sinensis* 10 g, *Pericarpium Citri Reticulatae* 9 g, *Rhizoma Cimicifugae* 12 g, and *Radix Bupleuri* 20 g; NONG’s granular preparation, dissolved in 200 mL warm boiled water and taken orally or by nasal feed once daily).

### Outcome measures

#### Laboratory tests

In both groups laboratory tests will include routine blood, urine, and fecal tests, blood gas analysis, liver and kidney function, coagulation function, and lactic acid, C-reactive protein, and procalcitonin analysis.

### Primary outcome

#### Efficacy evaluation criteria in TCM

Evaluation criteria by clinical manifestations are in reference to the *Criteria of Diagnosis and Therapeutic Effect of Diseases and Syndromes in Traditional Chinese Medicine* (Chinese Medicine Industry Standards of the People’s Republic of China; ZY/T001.1–94):

1. Clinical control: After treatment, symptoms and signs almost disappear and patients return to normal activities and work. The efficacy rate for syndromes is ≥ 90%.
2. Excellent efficacy: After treatment, symptoms and signs and the results of examinations improve significantly. The efficacy rate for syndromes is ≥ 60% but < 90%.
3. Efficacy: After treatment, symptoms and signs and the results of examinations improve. The efficacy rate for syndromes is ≥ 30% but < 60%.
4. Lack of efficacy: After treatment, symptoms and signs and the results of examinations do not improve compared to before treatment. The efficacy rate for syndromes is < 30%.

### Secondary outcomes

1. Clinical pulmonary infection score (CPIS): CPIS is a comprehensive clinical and imaging standard to assess the severity of pulmonary infection. It includes the following indicators: body temperature, white blood cell count, tracheal secretions, oxygenation, X-ray pulmonary infiltration, and progress in etiology. The clinical pulmonary infection score is compared before and after treatment. The CPIS details can be found in Additional file 3. 
2. Evaluation of disease severity: the scores of patients in the two groups are compared using the APECHA II scoring system before and after treatment. Improvement rate in APECHA II score = (Post-treatment score − Pre-treatment score)/Pre-treatment score. The APECHAII details can be found in Additional file 2.
3. Clearance of drug-resistant pathogens: observed on days 7, 14, 21, and 28.
4. Comparison of mechanical ventilation time and success rate of weaning off the ventilator are calculated if appropriate.
5. Comparison of mortality: mortality is compared on day 28 between the groups.
6. Radiography indicators: pulmonary CT imaging results are compared between the groups before and after treatment.
7. Number of hospitalization days: survival days of patients who die and hospitalization days of those who survive and are discharged.

### Data collection and management

**Fig. 2:**
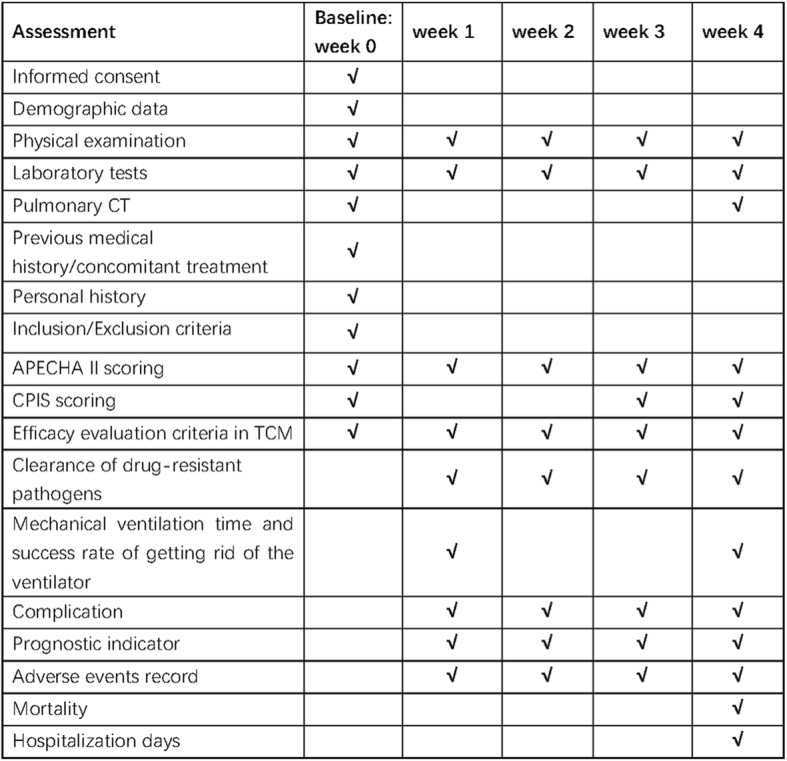
Standard Protocol Items: Recommendations for Interventional Trials (SPIRIT) flowchart

Data will be managed using the electronic data capture (EDC) system. The study protocol and case report form (CRF)-related materials are provided by the research team to a professional institution for clinical data management and study data statistics, which will establish an electronic case report form (eCRF) for the team, set up a personalized database for data recording, and is responsible for management of the study site’s EDC system. The Standard Protocol Items: Recommendations for Interventional Trials (SPIRIT) flowchart of the trial is shown in Fig. 2.

### Data monitoring

During the study, an independent data monitoring committee (DMC) will be set up to carry out periodic interim evaluation and optimize the study when appropriate based on the results of the interim evaluation. The DMC is authorized to discontinue the clinical study in case of unexpected adverse reactions. During implementation of the project, the original trial data will be subject to audit and random inspection periodically or irregularly and study compliance will be checked, so that data integrity and accuracy will be fully guaranteed and authenticity and reliability of the study results are ensured.

### Adverse events

Any untoward medical event that occurs in subjects during observation of a clinical study, whether or not it is causally related to the investigational drug, is considered an adverse event (AE). The AE report form is to be completed during the trial. The time of occurrence, severity, duration, actions taken, and outcomes of AEs are recorded. AEs occurring during follow-up should be reported in a timely manner to the sponsor; in case of serious AEs, the events should be reported to the adverse drug reactions (ADR) monitoring center of the local authority within 24 h, and to the sponsor at the same time.

### Sample size

Considering that no previous studies have been conducted to calculate the sample size, and assuming that the withdrawal rate is less than 15% [23], we expect that 100 patients will be enrolled in each arm, and that the final sample size of each arm will be at least 85. The data we collect will be helpful to calculate the appropriate sample size in the future and provide reference for further large-scale research.

### Statistical analysis

#### Data analysis

The Longhua Hospital Shanghai University of Traditional Chinese Medicine will be in charge of data management and statistical analysis in this study. Statistical analysts are not involved in clinical observation; they are responsible for statistical analysis of study data and timely delivery of statistical reports to the study director. Statistical analysis will be performed with SPSS 20.0 software. Measurement data are expressed as mean ± standard deviation. Normality test and homogeneity test of variance will be performed first. In case of normal distribution and homogeneity of variance, a *t*-test is performed, otherwise a non-parametric test is used. Count data are expressed as frequency constituent ratio (percentage) and *X*^*2*^ test is performed. *P* < 0.05 indicates statistical significance.

#### Consent

Patients who meet the study requirements will be offered a consent form covering the study name, registered information, research background, how the study is to be conducted, what participants should do in the study, inclusion/exclusion criteria, treatment plans and obligations, possible drug-related side effects, expenses during participation, etc. We will make every effort to protect the privacy of patients’ personal medical data to the extent permitted by law. Participation in this study is completely voluntary, and other treatment options will be offered to patients who do not participate or drop out. When patients sign the informed consent form, their personal and medical information will be used in this study.

#### Confidentiality

Participants’ medical records will be kept at the hospital, and the investigator, the research authority, and the ethics committee will be allowed access to these medical records. Any public report on the results of this study will not disclose participants’ personal identities. We will make every effort to protect the privacy of participants’ personal medical data to the extent permitted by law. Personal and medical information will be kept confidential in a safe and reliable place. At any time, participants may request access to their personal information (such as address, contact information, etc.) and may modify this information if necessary.

## Discussion

Infections caused by drug-resistant bacteria have become a major problem that seriously threatens the health of Chinese people and impacts national medical finance. It is of great scientific and clinical significance to actively carry out research on the prevention and treatment of MDRO infections. With the extensive use of broad-spectrum antibiotics in clinical practice, the problem of bacterial resistance is becoming more and more serious [24–26]. The *Enterobacteriaceae* in an Indian outbreak in 2010 were resistant to all the available antibiotics, which attracted global attention [27]. On January 29, 2018, the World Health Organization (WHO) published monitoring data on bacterial resistance to antibiotics for the first time, which revealed that bacterial resistance was widespread, with serious bacterial infections and high drug resistance in both high- and low-income countries, and had become a major public health problem worldwide [28].

By analyzing the clearance of drug-resistant pathogens, APECHA II score, CPIS score, TCM diagnostic and therapeutic criteria, mortality and related physical and chemical indicators, this study may confirm the clinical safety and efficacy of *Buzhong Yiqi* decoction in the treatment of MDRO HAP. In addition, by analyzing CRP, platelets, blood lactic acid, procalcitonin, and coagulation function, we will reveal the possible anti-inflammatory mechanism of *Buzhong Yiqi* decoction in treating MDRO HAP. Finally, if the hypothesis of this study is proved to be true, it can not only provide a clinical basis for the theory of “acute *Qi* deficiency” for HAP caused by MDRO, but also enrich the theory and practice of treating infectious diseases with TCM. Additonally, it is important to form a clinical diagnosis and treatment plan or guidance for comprehensive treatment of MDRO HAP with TCM, so as to reduce the medical and financial burdens caused by infections.

### Trial status

Protocol version 1, 11 February 2018.The recruitment commenced in July 2018 and aims to enroll 200 participants for the trial. It is anticipated that recruitment will end by June 2021.

## Supplementary information

**Additional file 1.** SPIRIT checklist.

**Additional file 2.** APECHA II scoring.

**Additional file 3.** CPIS scoring.

## Data Availability

Not applicable.

## Abbreviations

CPIS: Clinical pulmonary infection score
CRF: Case report form
eCRF: Electronic case report form
EDC: Electronic data capture
HAP: Hospital acquired pneumonia
ICU: Intensive care unit
MDRO: Multi-drug resistant organisms
TCM: Traditional Chinese medicine
VAP: Ventilatilator-associated pneumonia
