# Supplementary material for: Treatment of hospital-acquired pneumonia with multi-drug resistant organism by Buzhong Yiqi decoction based on Fuzheng Quxie classical prescription: study protocol for a randomized controlled trial
Source: Trials. 2019 Dec 30;20:817. doi: 10.1186/s13063-019-3927-x (PMC6937919; doi:10.1186/s13063-019-3927-x)
Supplement: Supplementary file 1 — Additional file 1. SPIRIT checklist. [file 13063_2019_3927_MOESM1_ESM.docx]

| SPIRIT CHECKLIST | |
| --- | --- |
| 1: Title | √ |
| 2: Trial registration | √ |
| 2a: Registry | √ |
| 2b: Data set | √ |
| 3: Protocol version | √ |
| 4: Funding | √ |
| 5: Roles and responsibilities | √ |
| 6: Background and rationale | √ |
| 7: Objectives | √ |
| 8: Trial design | √ |
| 9: Study setting | √ |
| 10: Eligibility criteria | √ |
| 11: Interventions | √ |
| 12: Outcomes | √ |
| 13: Participant timeline | √ |
| 14: Sample size | √ |
| 15: Recruitment | √ |
| 16: Allocation | √ |
| 17: Blinding (masking) | √ |
| 18: Data collection methods | √ |
| 19: Data management | √ |
| 20: Statistical methods | √ |
| 21: Data monitoring | √ |
| 22: Harms | √ |
| 23: Auditing | √ |
| 24: Research ethics approval | √ |
| 25: Protocol amendments | √ |
| 26: Consent or assent | √ |
| 27: Confidentiality | √ |
| 28: Declaration of interests | √ |
| 29: Access to data | √ |
| 30: Ancillary and post-trial care | √ |
| 31: Dissemination policy | √ |
| 32: Informed consent materials | √ |
| 33: Biological specimens | √ |
| Figure | √ |
| References | √ |
