# Supplementary material for: Treatment of hospital-acquired pneumonia with multi-drug resistant organism by Buzhong Yiqi decoction based on Fuzheng Quxie classical prescription: study protocol for a randomized controlled trial
Source: Trials. 2019 Dec 30;20:817. doi: 10.1186/s13063-019-3927-x (PMC6937919; doi:10.1186/s13063-019-3927-x)
Supplement: Supplementary file 2 — Additional file 2. APECHA II scoring. [file 13063_2019_3927_MOESM2_ESM.docx]

**Additional file 2: APECHA II scoring system**

| **A.age** | | ≤44□0；    45-54□2；    55-64□3；   65-74□5； ≥75□6 | | | | | | **A** **scoring** |  | | |
| --- | --- | --- | --- | --- | --- | --- | --- | --- | --- | --- | --- |
| **B.Chronic health score** | | | | After non-surgical or elective surgery □2；  Cannot be performed after surgery or emergency surgery □5；  None of the above □0. | | | | **B scoring** |  | | |
| **C.Acute physiological score** | | Points value | | | | | | | | | **score** |
|  |  | +4 | +3 | +2 | +1 | 0 | +1 | +2 | +3 | +4 |  |
| 1.Temperature (alar ℃) | | ≥41 | 39-40.9 |  | 38.5-38.9 | 36-38.4 | 34-35.9 | 32-33.9 | 30-31.9 | ≤29.9 |  |
| 2.Meam Blood Pressure  （mmHg） | | ≥160 | 130-159 | 110-129 |  | 70-109 |  | 50-69 |  | ≤49 |  |
| 3. Heart rate (beats/min) | | ≥180 | 140-179 | 110-139 |  | 70-109 |  | 55-69 | 40-54 | ≤39 |  |
| 4.Respiratory rate (per minute) | | ≥50 | 35-49 |  | 25-34 | 12-24 | 10-11 | 6-9 |  | ≤5 |  |
| 5.alternative | PaO2 （FiO2<50%） |  |  |  |  | ＞70 | 61-70 |  | 55-60 | ＜55 |  |
|  | A-aDO2（FiO2>50%） | ≥500 | 350-499 | 200-349 |  | ＜200 |  |  |  |  |  |
| 6.alternative | arterial blood PH | ≥7.7 | 7.6-7.69 |  | 7.5-7.59 | 7.33-7.49 |  | 7.25-7.32 | 7.15-7.24 | ＜7.15 |  |
|  | serum HCO3（mmol/L）  (for anemic) | ≥52 | 41-51.9 |  | 32-40.9 | 23-31.9 |  | 18-21.9 | 15-17.9 | ＜15 |  |
| 7.serum sodium（mmol/L） | | ≥180 | 160-179 | 155-159 | 150-154 | 130-149 |  | 120-129 | 111-119 | ≤110 |  |
| 8. serum potassium（mmol/L） | | ≥7 | 6-6.9 |  | 5.5-5.9 | 3.5-5.4 | 3-3.4 | 2.5-2.9 |  | ＜2.5 |  |
| 9. serum creatinine（μmol/L） | | ≥309.4 | 176.8-301 | 133-168 |  | 53-124 |  | ＜53 |  |  |  |
| 10. hematocrit (%) | | ≥60 |  | 50-59.9 | 46-49.9 | 30-45.9 |  | 20-29.9 |  | ＜20 |  |
| 11. white blood cell count (×10^9^/L) | | ≥40 |  | 20-39.9 | 15-19.9 | 3-14.9 |  | 1-2.9 |  | ＜1 |  |
| 12. neurological function（15-GCS） |  | Points value | | | | | | | | | |
|  |  | 6 | 5 | 4 | | 3 | | 2 | 1 | | GCS integral =1+2+3 |
|  | I.Eye Opening Response |  |  | □Automatically open | | □Call open eyes | | □Stabbing pain and opening eyes | □Unable to open eyes | |  |
|  | Ⅱ.  Language reaction |  | □Answer to the point | □The answer is beside the point | | □irrelevant answer | | □Only pronunciation | □Speechless | |  |
|  | Ⅲ.  Motion response | □Do as one is told | □The sting will locate | □The sting can be evaded | | □Pricking limb flexion | | □Stabbing limb extension | □Inactivity | |  |
| **C Score** | | | | | | | | | | |  |
| **APACHEⅡTotal score =A+B+C** | | | | | | | | | | |  |

Remarks:

1. Data acquisition should be the worst within 24 hours of admission to ICU or rescue.

2. Item B refers to the past history of severe organ system dysfunction or immunosuppression. The diagnostic criteria are: (1) liver: history of pathologically diagnosed cirrhosis and portal hypertension; upper gastrointestinal bleeding caused by portal hypertension; and previous liver failure/hepatic coma. (2) Cardiovascular system: New York Heart Association Cardiac Function Classification IV. (3) Respiration: COPD leads to severe activity limitation; secondary erythrocytosis; severe pulmonary hypertension (>40 mmHg); ventilator dependence. (4) Immunosuppression: treatment that inhibits anti-infective ability, such as immunosuppressive agents, chemotherapy, radiotherapy, recent and long-term use of hormones; serious progressive diseases that inhibit anti-infective ability, such as leukemia, lymphoma, AIDS.

3. In item B, "no operation" should be understood as a person who is unable to undergo surgical treatment because of his critical condition.

4. The blood pressure in item C should be mean arterial pressure= (systolic pressure + 2 x diastolic pressure)/3, and direct arterial pressure should be recorded if there is direct arterial pressure monitoring.

5. Respiratory frequency should be recorded.

6. If the patient is suffering from acute renal failure, the serum creatinine score should be doubled (*2)

7. When the unit of serum creatinine is micromol/L, the corresponding values with mg/dL are as follows:

mg/dL       3.5    2-3.4      1.5-1.9    0.6-1.4    0.6

μmol/L     305    172-304    128-171    53-127      53
