# Supplementary material for: Treatment of hospital-acquired pneumonia with multi-drug resistant organism by Buzhong Yiqi decoction based on Fuzheng Quxie classical prescription: study protocol for a randomized controlled trial
Source: Trials. 2019 Dec 30;20:817. doi: 10.1186/s13063-019-3927-x (PMC6937919; doi:10.1186/s13063-019-3927-x)
Supplement: Supplementary file 3 — Additional file 3. CPIS scoring. [file 13063_2019_3927_MOESM3_ESM.docx]

**Additional file 3: Clinical pulmonary infection score: (CPIS)**

| **Items** | **0 points** | **1 points** | **2 points** | **score** |
| --- | --- | --- | --- | --- |
| Average temperature (12 hours, ℃) | 36～38 | 38～39 | ＞39 or＜36 |  |
| white blood cell count (×109/L) | 4～11 | 11～17 | ＜4 or＞17 |  |
| Secretions (number of 24 hour aspirates) | No phlegm or a little | Medium to large, nonpurulent | Medium to large, purulent |  |
| Gas exchange index (PaO2/FiO2,kPa) | ＞33 |  | ＜33 |  |
| Chest X - ray infiltrates the image | nothing | patchy | Fusion flake |  |
| Tracheal aspirate culture or sputum culture | No pathogenic bacteria growth | Existence of pathogenic bacteria growth | Two cultures of the same bacteria or Gran staining were consistent with the culture. |  |

Remarks: 1. The highest score is 12 points, the higher the score, the worse the condition, the lower the score, and the remission of the condition;

2. When the score is less than 6 points, antibiotics can be discontinued.
